# Supplementary material for: On reactive Ion Etching of Parylene-C with Simple Photoresist Mask for Fabrication of High Porosity Membranes to Capture Circulating and Exfoliated Tumor Cells
Source: Micromachines (Basel). 2024 Apr 13;15(4):521. doi: 10.3390/mi15040521 (PMC11051955; doi:10.3390/mi15040521)
Supplement: Supplementary file 1 [file micromachines-15-00521-s001.zip › micromachines-2926433-supplementary.pdf]

micromachines

Supplementary Materials

S1. Porosity of close-packed hexagonal-shaped micropore array

A unit cell of the pore array is highlighted in **Fig. S1**. The pore area within the unit is 3x of an individual hexagonal pore. The porosity can be calculated as:

$$Porosity = \frac{3*A_{pore}}{A_{unit\ cell}} = 3*\left(\frac{D}{D'}\right)^2 = 3*\left[\frac{D}{2*(D+S)*\sin 60^o}\right]^2 = \left(\frac{D}{D+S}\right)^2, \quad (S1)$$

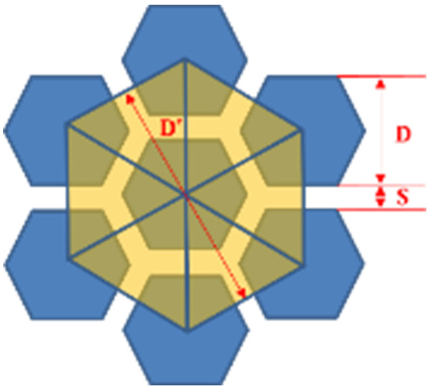

Figure S1. Schematic for porosity calculation of close-packed hexagonal-shaped micropore arrays.

Table S1. Membrane porosity for different array design.

S(μm) \ D(μm)	8	10	12	40
**4**	44.4%	51.0%	56.3%	N/A
**6**	32.7%	39.1%	44.4%	75.6%

Table S1 lists the nominal porosity of the membrane for different array design. The final fabricated membranes had porosity over 40% in general due to lateral etching of the pore-to-pore spacing during fabrication.

S2. Device and setup for capturing spiked tumor cells

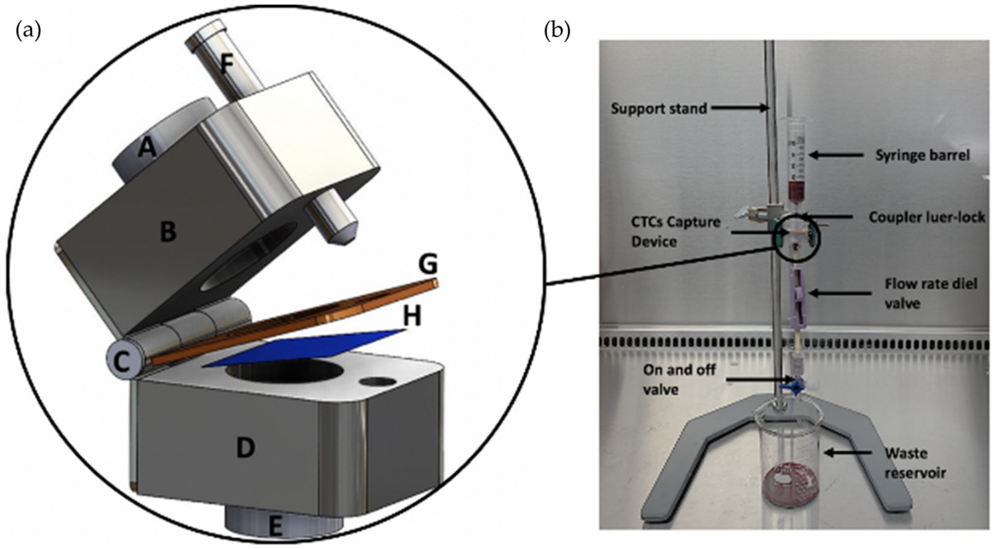

Figure S2. Device and setup for capturing spiked tumor cells. (a) Schematic of CTC Capturing Device. A: fluid inlet and reservoir lure-lock attachment, B: upper polycarbonate housing, C: flexible hinge, D: lower polycarbonate housing, E: waste outlet to flow rate control valve, F: device locking rod, G: rubber gasket (silicon 20A), H: parylene microporous membrane. (b) An image of experimental setup for CTC capture with a syringe barrel at the inlet, a flow regulator and shut-off valve at the outlet.

S3. Viability of captured SJSA-1 cells

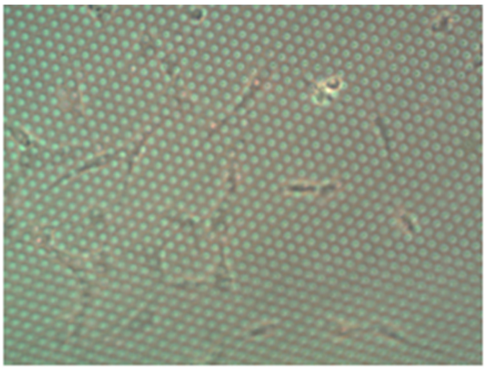

Figure S3. Viability of captured SJSA-1 cells: phase-contrast images showing growth of captured SJSA-1 cells on the Parylene-C membrane cultured inside a CO_2 incubator.
